# Current State of Developmental Neurotoxicology Research

**DOI:** 10.3390/toxics3040370

**Published:** 2015-10-01

**Authors:** David R Wallace

**Affiliations:** Department of Pharmacology & Physiology, Oklahoma State University Center for Health Sciences, 1111 West 17^th^ Street, Tulsa, OK, USA; E-Mail: david.wallace@okstate.edu; Tel.: +1-918-561-1407; Fax: +1-918-561-5729

We have been witness to significant research advances in areas such as neuroscience, neurodegeneration, cancer therapy, *etc.*, yet, investigation in developmental neurotoxicology (DNT) has fallen behind [1]. Reasons for this lag include; complexity in translating model systems to the human condition, the sensitivity of the developing brain to numerous xenobiotics, difficulty in performing the necessary toxicological testing on thousands of chemicals with incomplete toxicity profiles, and the complex nature of exposure to multiple agents simultaneously [2,3]. Independently, two or more compounds may be non-toxic, yet as a mixture; potentiating or synergistic toxic effects are observed. Areas with significant gaps in knowledge include; identifying appropriate model systems to study DNT effects to improve translation from non-human to human model systems [4], identification of accurate biomarkers that signal exposure to DNT compounds early in development, facilitating medical intervention. The goal of this special edition is to provide a broad overview into current work that is being performed in DNT. Presentations in this special edition examine and discuss DNT (both peripheral and neural) from aquatic biota toxicity to the gender-dependency of DNT following exposure to environmental toxins/pollutants.

Dr. Weis discusses the ecological effects of multiple environmental toxins (heavy metals, pesticides, polycyclic aryl hydrocarbons (PAH)) on developmental deficits observed in fish and other invertebrates [5]. In many instances, these deficits are delayed from initial exposure, and then perpetuated over generations which increase the difficulty in predicting developmental toxicity onset and progression. Ecological effects can be translated to similar responses observed in mammals. Ashworth *et al.* describe the influence of gender and C57BL/6 (B6) substrain on the outcomes of neurobehavioral studies [6]. Use of animal models has revealed the need for considering gender variations and the use of the appropriate testing system for accurately assessing developmental deficits observed following exposure toxins. An increasingly popular model system for studying DNT is the zebra fish. Lee and Freeman discuss the zebra fish as a model system for investigating DNT [7]. The ability to visualize synaptogenesis and neuronal growth during development makes the zebra fish an excellent model system to study both the environmental and the ecological effects of pollutants. Increasing evidence suggest that it results observed with zebra fish can also be translated to the human condition. Utilization of adult stem cells in addition to embryonic stem cells has expanded our ability to employ molecular modeling and smart design to develop a model system specifically suited for the needs of the investigator. Pallocca *et al.* discusses work with human embryonic stem cells and carcinoma pluripotent stem cells and the ability of methyl mercury to alter micro RNA (miRNA) expression in these cells [8]. Interference in appropriate miRNA expression can impact many cellular actions such as neurogenesis, differentiation, neurite outgrowth, and synaptogenesis. Harry *et al.* describe the effects of the dioxin-like compound, 3,3',4,4'-tetrachloroazobenzene (TCAB) exposure on the disruption of thyroid hormone function in rats [9]. Exposure to TCAB resulted in a dose-dependent reduction in thyroid (T4) hormone reduction. Thyroid hormone is vital for normal neuronal development during gestation and disruption of this hormone will lead to altered hippocampal arbor formation. Their work underscores the necessity of examining neuronal structures in addition to neurochemistry. Lastly, directly studying human responses bypasses the potential difficulty translating effects *in vitro*, or in a different species to the effects observed in humans. Exposure to multiple environmental agents simultaneously adds a significant level of complexity to the interpretation of human responses. De Felice *et al.* describe the multifactorial etiology of DNT affecting children following exposure to environmental chemicals as a risk factor for neurodevelopment [10]. They introduce the “exposome” concept and discuss how the human response is a composite of all exposures, risk factors, and socioeconomic variables a person is exposed to as part of their environment. They also discuss the need for more realistic models and accurate biomarkers to further this area of study.

Collectively, the articles in this special edition offer exciting insights into the present state of development toxicology. They cover many of the major focus points and describe strengths and weaknesses associated with each. Together they begin to pull the field together and provide direction for the future. There are three reviews and three original research articles which clearly demonstrate the diverse field of developmental neurotoxicology and I would like to thank each of the authors for their contributions to this special edition. It has been an honor and privilege to serve in the capacity of Guest Editor. I would like to express my gratitude to all of the reviewers who contributed their time and effort assisting in the completion of this edition. I also would like to thank Dr. Bellinger, Editor-in-Chief, Ms. Zu Qiu, Assistant Editor, and the entire *Toxics* Editorial Office for assisting me through the process.
